# Hydrogen ventilation combined with mild hypothermia improves short-term neurological outcomes in a 5-day neonatal hypoxia-ischaemia piglet model

**DOI:** 10.1038/s41598-019-40674-8

**Published:** 2019-03-11

**Authors:** Yinmon Htun, Shinji Nakamura, Yasuhiro Nakao, Tsutomu Mitsuie, Makoto Nakamura, Satoshi Yamato, Wataru Jinnai, Kosuke Koyano, Kenichi Ohta, Aya Morimoto, Takayuki Wakabayashi, Masashiro Sugino, Kazumichi Fujioka, Ikuko Kato, Sonoko Kondo, Saneyuki Yasuda, Takanori Miki, Masaki Ueno, Takashi Kusaka

**Affiliations:** 1 Graduate School of Medicine, Faculty of Medicine, Kagawa University, Kagawa, Japan; 2 Department of Pediatrics, Faculty of Medicine, Kagawa University, Kagawa, Japan; 3 Department of Neonatology, National Hospital Organization Okayama Medical Center, Okayama, Japan; 4 Division of Neonatology, Shikoku Medical Center for Children and Adults, Kagawa, Japan; 5 Maternal and Perinatal Center, Kagawa University Hospital, Kagawa, Japan; 6 Department of Anatomy and Neurobiology, Faculty of Medicine, Kagawa University, Kagawa, Japan; 7 Department of Pediatrics, Kobe University Graduate School of Medicine, Kobe, Japan; 8 Department of Pathology and Host Defense, Faculty of Medicine, Kagawa University, Kagawa, Japan

## Abstract

Despite its poor outcomes, therapeutic hypothermia (TH) is the current standard treatment for neonatal hypoxic-ischaemic encephalopathy (HIE). In this study, due to its antioxidant, anti-inflammatory, and antiapoptotic properties, the effectiveness of molecular hydrogen (H_2_) combined with TH was evaluated by means of neurological and histological assessments. Piglets were divided into three groups: hypoxic-ischaemic insult with normothermia (NT), insult with hypothermia (TH, 33.5 ± 0.5 °C), and insult with hypothermia with H_2_ ventilation (TH-H_2_, 2.1–2.7%). H_2_ ventilation and TH were administered for 24 h. After ventilator weaning, neurological assessment was performed every 6 h for 5 days. On day 5, the brains of the piglets were harvested for histopathological analysis. Regarding the neurological score, the piglets in the TH-H_2_ group consistently had the highest score from day 2 to 5 and showed a significantly higher neurological score from day 3 compared with the NT group. Most piglets in the TH-H_2_ group could walk at day 3 of recovery, whereas walking ability was delayed in the two other groups. The histological results revealed that TH-H_2_ tended to improve the status of cortical gray matter and subcortical white matter, with a considerable reduction in cell death. In this study, the combination of TH and H_2_ improved short-term neurological outcomes in neonatal hypoxic-ischaemic piglets.

## Introduction

Intrapartum-related hypoxic events, also known as birth asphyxia, result in varying degrees of neurological impairment and negatively impact a child’s long-term potential^1^. One of the severe consequences of birth asphyxia is hypoxic-ischaemic encephalopathy (HIE), which has a wide clinical spectrum that can include mental retardation^2^. The pathophysiology of HIE is highly complex. It is regarded as an evolving injury in which the primary phase of cell damage results from hypoxic-ischaemic (HI) events involving a rapid energy depletion. Following the return of cerebral circulation, the cytotoxic effects are briefly resolved (latent phase). After the latent phase, neurons undergo deterioration due to an accumulation of excitotoxins, which culminates in neuronal death^3–5^.

Inflammation and oxidative stress are generally considered to be the two major causes of cell death after ischaemic brain injury in neonatal HIE^6^. Therefore, alleviation of inflammation and elimination of oxidative stress are critical to prevent the cell death in neonatal HIE. The current standard treatment for HIE is therapeutic hypothermia (TH, mild hypothermia of 33–34 °C for 72 h). The possible effectiveness of TH has been extensively reported in both adult and paediatric medicine, including the neonatal field, mainly in terms of neuroprotection and cardioprotection. Many trials have shown that TH improves neurological outcomes after cardiac arrest in adults^7^. TH is also beneficial in term and late-preterm newborns with HIE, reducing mortality without increasing major disabilities^8^. However, for some conditions such as traumatic brain injury, stroke in adults and cardiac arrest in the paediatric population, its effectiveness remains unclear and warrants further investigation^9–11^. TH significantly reduces the combined rate of death and severe disability at 18-month follow-up^12^. However, secondary outcomes in later childhood reveal limited improvements with TH versus standard care^8,13,14^. Thus, to further improve outcomes, combination therapy with other neuroprotective agents has increasingly become a research focus. In the present study, we focused on the effectiveness of molecular hydrogen (H_2_) combined with TH.

H_2_ is regarded as an antioxidant, anti-inflammatory, and antiapoptotic agent that acts as a therapeutic and preventive antioxidant by selectively reducing the levels of highly active oxidants, such as hydroxyl radical (•OH) and peroxynitrite (ONOO^−^), in cultured cells^15^. It reduces oxidative stress directly by scavenging free radicals and indirectly by modulating the signalling pathways involved in inflammation, preventing damage to the cells and ultimately protecting them from further necrotic and apoptotic cell death^15,16^. Another advantage is that it passes through the blood–brain barrier, unlike most antioxidative agents, with minimal adverse effects on the human body^17^. Due to such properties, H_2_ has been extensively studied under physiological and pathological conditions and found to be effective^16^. Many studies of animals with ischaemic brain injury show improved neurological recovery after H_2_ treatment^15,18–21^.

Various studies have reported H_2_ ventilation-induced neuroprotection in neonatal animal models^18,20–22^ (Table 1). However, there are few studies of the effectiveness of TH-H_2_ in a large neonatal animal model that displays a functional improvement that is clinically applicable to human neonates.

**Table 1:** Summary of previous studies showing the effectiveness of hydrogen inhalation using neonatal and adult animal models (P = post-natal day).

	Animal model	Method of administration	Outcomes
Nemeth *et al*. (2016)	<1-day-old piglets	Inhalation of 2.1% H_2_ for 4 h (24-h survival)	Enhanced recovery of EEG, significant preservation of neurons, and reduction of oxidative markers
Hayashida *et al*. (2014)	Adult rats	Inhalation of 1.3% H_2_ for 2 h (7-day survival)	Rescued neuronal death and suppressed microglia activation in the hippocampus and cerebral cortex Improved animal survival and neurological recovery in post-cardiac arrest rats
Oláh *et al*. (2013)	1–2-day-old piglets	Inhalation of 2.1% H_2_ for 4 h (24-h survival)	Recovery of EEG function, modest neuroprotection in histopathology, and alleviated delayed neurovascular dysfunction
Matchett *et al*. (2009)	P10 rats	Inhalation of 2.9% H_2_ for 4 h (24-h survival)	Did not ameliorate moderate-to-severe ischaemic damage
Cai *et al*. (2008)	P7 rats	Inhalation of 2% H_2_ for 30, 60 or 120 min (24-h survival)	Provided brain protection in mild insult via inhibition of neuronal apoptosis in a duration-dependent manner
Ohsawa *et al*. (2007)	Adult rats	Inhalation of 1%, 2% or 4% H_2_ for 120 min (12-h, 3-day, and 7-day survival)	Oxidative markers substantially reduced in H_2_-treated rats and a distinct H_2_-dependent decrease in the accumulation of microglia

In this study, our unique neonatal HI piglet model was used, in which an HI insult was controlled and monitored both by amplitude-integrated EEG (aEEG) and by time-resolved near-infrared spectroscopy (TRS) monitoring of cerebral hemodynamics. Using this piglet model, we examined the effectiveness of H_2_ ventilation combined with TH in neonatal HI piglets via neurological and histological evaluations.

## Results

Twenty-four piglets were studied, distributed among the following three groups: HI insult with normothermia (NT, n = 9), HI insult with TH (TH, 33.5 ± 0.5 °C, n = 8), and HI insult with TH with H_2_ ventilation (TH-H_2_, 2.1–2.7% H_2_, n = 7). Two piglets, one from the NT group and another from the TH-H_2_ group, died within 5 days after the HI insult due to complications such as seizures, despite anticonvulsant therapy. Thus, data from the surviving piglets (n = 22; NT, n = 8, TH, n = 8, TH-H_2_, n = 6) were analysed.

### Physiological and arterial blood gas data

There were no significant differences among the three groups in heart rate (HR), mean arterial blood pressure (MABP), or rectal temperature (RT) at baseline (Table 2). All three groups had a significant reduction in HR and MABP at the end of insult (0 h) that gradually returned to baseline.

**Table 2:** Physiological parameters at baseline, 0 h (end of insult), 1 h, 6 h, 12 h, and 24 h after the insult.

Parameters		Baseline	0 h	1 h	6 h	12 h	24 h
HR (bpm)	NT	211.6 ± 29.9	146.8 ± 19.4****	233.3 ± 24.8	238.8 ± 14.5^#^	237.6 ± 22.5^#^	203.3 ± 30.9
	TH	214.8 ± 42.0	172.8 ± 42.0*	202.4 ± 36.8	213.9 ± 7.4^#^	210.4 ± 14.0^#^	185.4 ± 4.2
	TH-H_2_	215.2 ± 10.9	160.2 ± 21.8**	234.3 ± 24.9	182.2 ± 21.9	176.8 ± 30.1	177.0 ± 23.2
MABP (mmHg)	NT	79.0 ± 7.0	47.3 ± 13.3****	62.6 ± 7.0**	70.0 ± 11.9	69.8 ± 8.3	62.1 ± 8.9**
	TH	79.5 ± 16.1	51.3 ± 10.9****	74.9 ± 6.3	70.3 ± 8.3	69.3 ± 8.3	63.0 ± 8.5*
	TH-H_2_	73.2 ± 9.6	49.3 ± 14.0***	68.0 ± 2.9	74.7 ± 8.3	68.7 ± 8.8	65.0 ± 4.4
RT (°C)	NT	37.7 ± 0.7	37.7 ± 0.6	38.2 ± 0.6^#^	38.5 ± 0.3^*,#^	37.9 ± 0.5^#^	38.4 ± 0.5^#^
	TH	36.8 ± 0.7	36.8 ± 0.8	33.7 ± 0.8^****,#^	33.9 ± 0.3****	34.0 ± 0.4^****,#^	34.2 ± 0.4****
	TH-H_2_	38.2 ± 0.8	37.5 ± 0.9	36.4 ± 1.1	32.8 ± 1.3****	32.9 ± 0.9****	35.2 ± 1.6**

Values are expressed as mean ± SD. NT indicates normothermia; RT, rectal temperature; TH, therapeutic hypothermia and TH-H_2_, therapeutic hypothermia with hydrogen ventilation. *p < 0.05, **p < 0.01, ***p < 0.001, ****p < 0.0001 versus baseline; ^#^p < 0.05 versus TH-H_2_.

Biochemical parameters such as PaO_2_, PaCO_2_, pH, base excess, lactate, glucose, and haemoglobin at baseline showed no significant differences among the three groups (Table 3). pH, PaO_2_ and base excess were significantly reduced at the end of insult (0 h) and blood lactate was significantly higher at the end of insult in the three groups compared with their respective baseline values. pH at 1 h after the insult was lowest in the TH group. The base excess was significantly acidotic in the NT group at 6, 12 and 24 h after the insult. PaCO_2_ was relatively maintained at a constant value for 24 h after the insult.

**Table 3:** Arterial blood gas data at baseline and 0 h (end of insult), 1 h, 6 h, 12 h, and 24 h after the insult.

Parameters		Baseline	0 h	1 h	6 h	12 h	24 h
pH	NT	7.42 ± 0.05	6.85 ± 0.09****	7.30 ± 0.06**	7.46 ± 0.04	7.46 ± 0.05^#^	7.50 ± 0.05^*,#^
	TH	7.44 ± 0.11	6.79 ± 0.09****	7.22 ± 0.10^****,#^	7.45 ± 0.05	7.44 ± 0.05^#^	7.43 ± 0.04^#^
	TH-H_2_	7.43 ± 0.03	6.90 ± 0.09****	7.36 ± 0.08	7.40 ± 0.04	7.37 ± 0.04	7.35 ± 0.05
pCO_2_ (mmHg)	NT	46.9 ± 4.6	34.5 ± 10.6**	42.2 ± 6.2	47.5 ± 5.9	45.1 ± 4.6	39.3 ± 3.1
	TH	41.6 ± 11.8	44.8 ± 12.8	43.8 ± 8.7	41.9 ± 7.0	40.9 ± 6.7	36.4 ± 5.3
	TH-H_2_	43.0 ± 3.4	39.2 ± 11.6	37.8 ± 3.4	43.7 ± 4.7	43.8 ± 2.0	42.4 ± 6.6
pO_2_ (mmHg)	NT	89.6 ± 10.8	17.5 ± 6.7****	94.5 ± 26.5	89.3 ± 9.4	85.8 ± 14.2	89.7 ± 14.1
	TH	98.3 ± 13.7	19.7 ± 6.3****	115.4 ± 22.3	82.8 ± 22.1	83.7 ± 23.2	81.4 ± 20.5
	TH-H_2_	104.5 ± 4.4	26.2 ± 18.1****	121.7 ± 23.1	105.0 ± 18.7	103.5 ± 17.7	109.8 ± 29.0
BE (mmol/L)	NT	5.8 ± 2.3	−26.0 ± 4.7****	–5.5 ± 3.7****	8.6 ± 1.5^#^	7.2 ± 3.2^#^	6.8 ± 2.5^#^
	TH	3.1 ± 3.0	−27.3 ± 31****	–9.8 ± 4.3****	4.5 ± 2.9	3.5 ± 3.0	0.0 ± 2.7
	TH-H_2_	3.6 ± 2.0	−22.5 ± 3.2****	–3.8 ± 4.2**	2.2 ± 3.6	0.7 ± 2.8	–2.1 ± 3.8*
Lactate (mg/dL)	NT	18.0 ± 4.1	227.1 ± 26.2^****,#^	122.5 ± 28.4****	27.8 ± 9.2	36.9 ± 11.0	34.4 ± 9.7
	TH	20.0 ± 8.3	213.5 ± 23.7^****,#^	121.9 ± 26.3****	33.3 ± 14.4	39.4 ± 14.4	49.5 ± 11.4*
	TH-H_2_	16.8 ± 2.2	172.8 ± 22.8****	91.5 ± 17.8****	32.7 ± 6.2	38.8 ± 5.9	56.7 ± 24.0**
Glucose (mg/dL)	NT	146.0 ± 18.1	253.5 ± 68.5**	237.0 ± 53.3*	183.9 ± 53.1	195.5 ± 67.9	115.6 ± 37.5
	TH	155.8 ± 37.6	230.8 ± 97.7	256.5 ± 65.5*	219.4 ± 66.9	222.1 ± 68.7	191.0 ± 48.3
	TH-H_2_	147.5 ± 18.8	231.3 ± 62.1*	204.0 ± 51.0	194.7 ± 40.8	230.0 ± 22.5*	179.2 ± 67.9

Values are expressed as mean ± SD. NT indicates normothermia; TH, therapeutic hypothermia and TH-H_2_, therapeutic hypothermia with hydrogen ventilation. *p < 0.05, **p < 0.01, ***p < 0.001, ****p < 0.0001 versus baseline; ^#^p < 0.05 versus TH-H_2_.

### Time to reach the target temperature after TH

The piglets in this study were under anaesthesia-ventilation for 24 h after the HI insult. The mean time ± standard deviation (SD) to reach the target temperature (34 °C) was 84.3 ± 43.6 min in the TH group and 99.3 ± 42.4 min in the TH-H_2_ group. There was no statistically significant difference between the two groups.

### Electrocortical activity

The total duration of low-amplitude-integrated EEG (LAEEG) during and after the insult was not statistically significant among the three groups: NT, 46.4 ± 13.0 min, TH, 53.9 ± 12.2 min, and TH-H_2_, 50.2 ± 15.8 min.

### Neurological score

**Figure 1:**
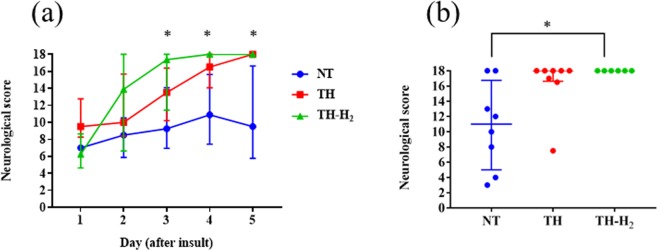
(**a**) Neurological scores from day 1 to day 5 (median with interquartile range). The TH-H_2_ group had a significant improvement in the neurological score from day 3 post-insult compared with the NT group (p < 0.05). (**b**) Neurological score at day 5 (median with interquartile range). All piglets in the TH-H_2_ group reached the full score of 18 at day 5 (p < 0.05 versus the NT group).

**Figure 2:**
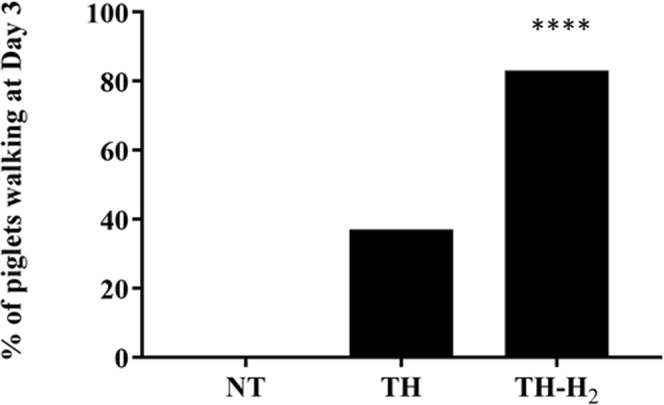
Percentage of piglets walking on day 3. None of the piglets in the NT group could walk on day 3 after the insult (0%). Three of the eight piglets (37.5%) in the TH group were walking on day 3 post-insult and 5 of the 6 piglets (83%) in the TH-H_2_ group were walking on day 3 post-insult. The piglets in the NT group regained normal walking function after day 3 or were unable to walk until day 5. In the TH-H_2_ group, the earliest recovery of walking function was seen on day 1 post-insult (p < 0.0001).

The neurological score tended to increase from day 1 to day 5 in the TH and TH-H_2_ groups but was lower on day 5 than on day 4 in the NT group. On day 1, the neurological score was higher in the TH group than in the NT and TH-H_2_ groups. However, from day 2 onwards, the neurological score of the TH-H_2_ group rapidly increased, eventually surpassing that of the TH group, whose score was initially higher. TH-H_2_ continued to maintain a higher score until day 5. In addition, the neurological scores on day 3, day 4 and day 5 were significantly higher in the TH-H_2_ group than in the NT group (Fig. 1a,b). The median neurological scores (interquartile range) in the NT, TH, and TH-H_2_ groups on day 5 were 9.5 (5.8–16.6), 18.0 (16.6–18.0) and 18 (18.0–18.0), respectively. Regarding ability to walk, none of the 8 piglets was able to walk in the NT group on day 3, whereas 3 of the 8 piglets (37.5%) were walking in the TH group and 5 of the 6 piglets (83%) were walking in the TH-H_2_ group on day 3 (p < 0.0001) (Fig. 2). Normal and abnormal patterns of walking movements of some piglets were video recorded (Supplementary information).

### Histological scores

**Figure 3:**
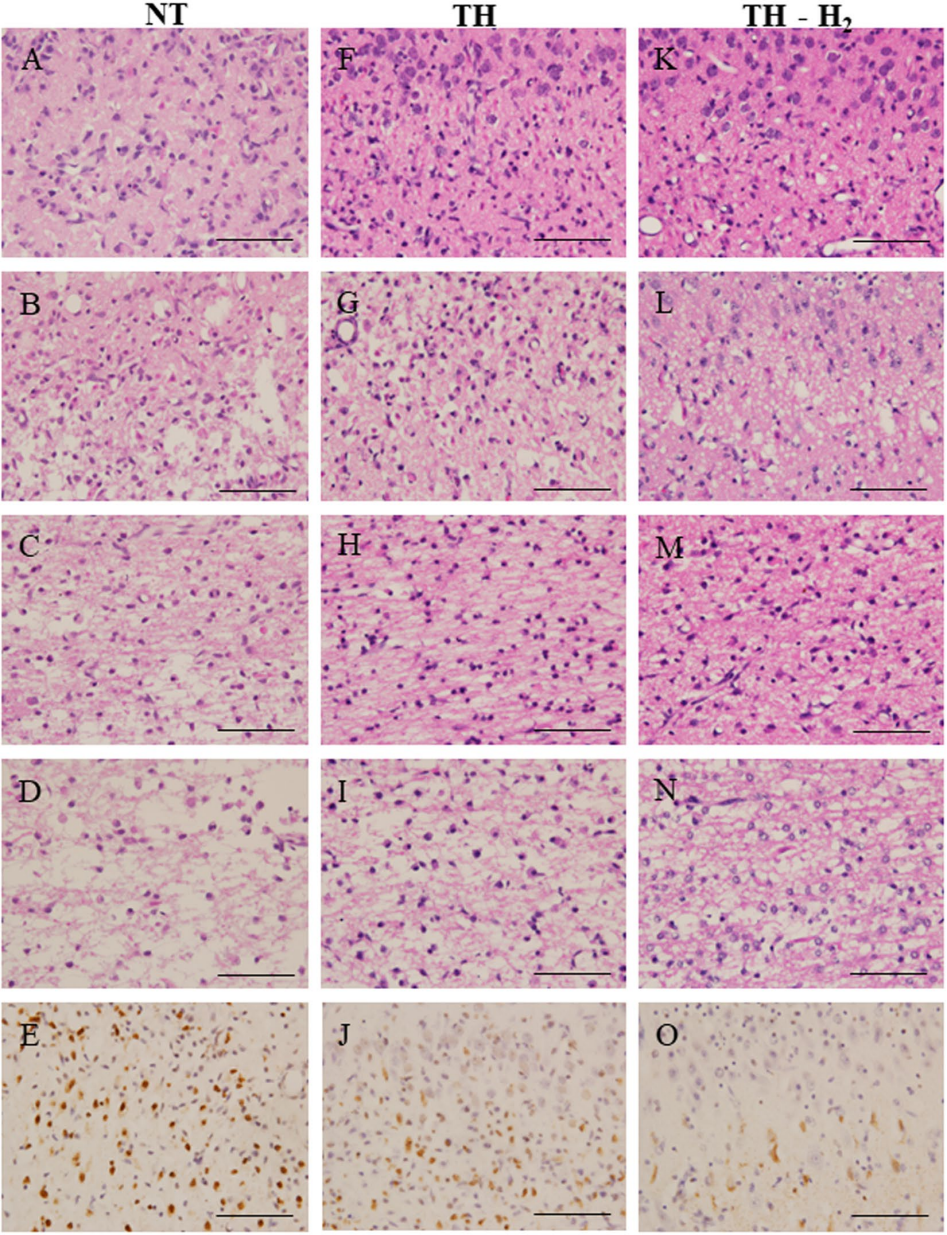
Representative images of haematoxylin & eosin (H&E) staining (**A**–**D**, **F**–**I**, and **K**–**N**) and TUNEL staining (**E**,**J**,**O**) in the dorsal cerebral cortex in the NT (**A**–**E**), TH (**F**–**J**), and TH-H_2_ (**K**–**O**) groups. Two representative images of H&E staining indicating weaker (**A**,**C**,**F**,**H**,**K**,**M**) and stronger (**B**,**D**,**G**,**I**,**L**,**N**) tissue damage are shown for each group of gray (**A**,**B**,**E**,**F**,**G**,**J**,**K**,**L,O**) and white (**C**,**D**,**H**,**I**,**M**,**N**) matters of the cerebral cortex. Scale bars indicate 100 μm.

Four areas of the brain of the piglet is studied; cortical gray matter, subcortical white matter, hippocamous and cerebellum. In hematoxylin and eosin (H&E) score, the values are expressed in median (interquartile range). According to H&E scores, tendency of improvement was seen in gray matter and white matter of TH-H_2_ group. The scores are ranging from 3.3 (1.6–3.9) in NT, 2.2 (1.3–3.5) in TH and 1.8 (0.4–2.7) in TH-H_2_ respectively. Haematoxylin & eosin (H&E) staining of the gray matter of the cerebral cortex of the NT group revealed that most pyramidal neurons were affected by infiltration of inflammatory cells and that a considerable number of neurons had become necrotic in some animals (Fig. 3A). In other animals of the group, many clear necrotic neurons with a pyknotic nucleus and eosinophilic cytoplasm were seen together with neuropil showing severe vacuolar degeneration and infiltration of numerous inflammatory cells (Fig. 3B). In the gray matter of the cerebral cortex of the TH group, some pyramidal neurons were spared, although many necrotic neurons were seen in some animals (Fig. 3F) and most neurons were deteriorated or had become necrotic with degenerative neuropil in other ones (Fig. 3G). In the gray matter of the cerebral cortex of the TH-H_2_ group, a considerable number of pyramidal neurons was spared, although some necrotic neurons were seen in some animals (Fig. 3K) and a considerable number of necrotic neurons and some spared neurons showing a pyramidal structure were seen with degenerative neuropil in other ones (Fig. 3L).

**Figure 4:**
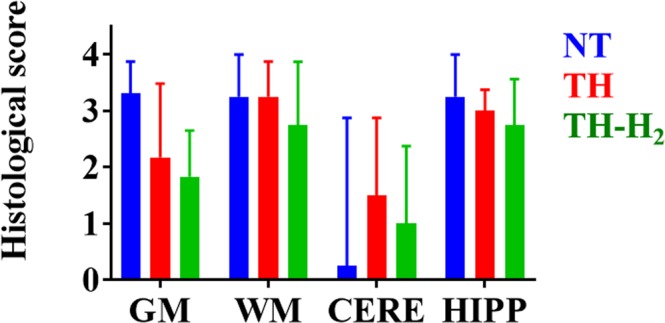
Histological scoring using hematoxylin and eosin staining in cortical gray matter (GM) and subcortical white matter (WM), the hippocampus (HIPP), and the cerebellum (CERE). There was a tendency for less damage in the GM and WM in the TH-H_2_ group compared with the NT group (median with interquartile range).

The damage in white matter appears to be more severe in this model. The scores of all three groups are relatively higher compared to other parts of the brain examined. NT group; 3.3 (3-4), TH group 3.3 (0.6–3.9) and TH-H_2_ group 2.8 (0.8–3.9) respectively. H&E staining of the white matter of the cerebral cortex of the NT group revealed moderate oedematous and vacuolar degeneration in neuropil in most areas of some animals (Fig. 3C), whereas completely deteriorated tissue showing no neuronal fibre structure with infiltration of some inflammatory cells was seen in other ones (Fig. 3D). In the white matter of the cerebral cortex of the TH group, mildly oedematous neuropil with infiltration of inflammatory cells was seen in some animals (Fig. 3H), whereas moderately oedematous neuropil with degenerative fibre structure and infiltration of inflammatory cells was seen in other ones (Fig. 3I). In the white matter of the cerebral cortex of the TH-H_2_ group, mildly oedematous neuropil with infiltration of inflammatory cells was seen in some animals (Fig. 3M), whereas mild-to-moderate oedematous neuropil with partially spared fibre structure and infiltration of inflammatory cells was seen in other ones (Fig. 3N). Cortical gray matter and subcortical white matter of TH-H_2_ group showed lower histological score on H&E (Fig. 4).

**Figure 5:**
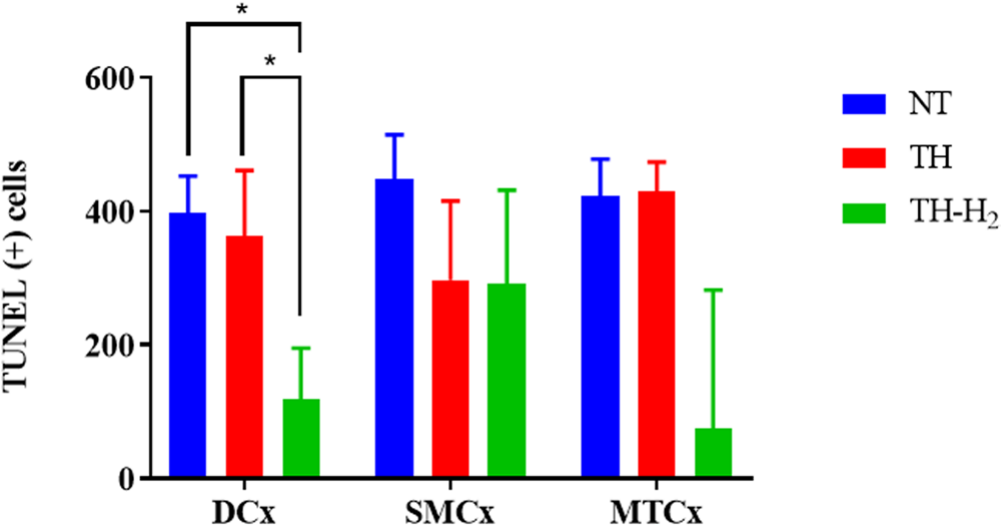
Number of TUNEL (+) cells in three regions of the cerebral cortex, namely, the dorsal cortex (DCx), sensorimotor cortex (SMCx), and mid-temporal cortex (MTCx). In the DCx, significantly fewer TUNEL (+) cells were seen in the TH-H_2_ group compared with the NT and the TH groups (median with interquartile range). A p value of < 0.05 was considered statistically significant.

Terminal deoxynucleotidyl transferase-mediated dUTP nick-end labelling (TUNEL) revealed that many of the remaining degenerative neuronal cells of the NT group showed TUNEL-positive (+) staining in the cerebral cortex (Fig. 3E). In the cerebral cortex of the TH group, a considerable number of degenerating neuronal cells showed TUNEL (+) staining, whereas some remaining pyramidal neurons showed no staining (Fig. 3J). In the cortex of the TH-H_2_ group, some degenerative cells showed TUNEL (+) staining, whereas the remaining pyramidal neurons showed no staining (Fig. 3O). The TH-H_2_ group had significantly fewer TUNEL (+) cells in the dorsal cortex (DCx) compared with the NT and TH groups (Fig. 5). The number of TUNEL (+) cells in DCx are, NT: 396.5 (259.8–452.8), TH: 363.5 (212.3–461.0) and TH-H_2_: 118.5 (56.3–195.3) respectively. The tendency of improvement was seen in TH-H_2_ group, however, there was no statistical significance in sensorimotor cortex (SMCx) and mid-temporal cortex (MTCx) among three groups in TUNEL staining. The values are expressed in median (interquartile range). A p value < 0.05 was considered statistically significant.

## Discussion

This study is the first to examine the effectiveness of H_2_ ventilation combined with TH in neonatal HI piglets over a 5-day period. In our model, compared with NT, TH-H_2_ significantly improved short-term neurological outcomes from day 3 after the insult, with a higher percentage of piglets recovering the ability to walk. However, histological improvement was limited, with TH-H_2_ only improving the gray matter and white matter, but not the hippocampus or cerebellum. In TUNEL staining, the number of TUNEL (+) cells was significantly lower in the gray matter of the dorsal cortex of the TH-H_2_ group compared with the other groups.

The effective neuroprotection induced by combination therapy in this study could be due to the prevention of further cell death by H_2_, which augments the neuroprotective effects of TH initiated within 6 h after the insult (possible latent phase). Although cell death due to primary HI insult was not preventable, the delayed cell death of both necrosis and apoptosis appeared to be reduced in this study. In one study of a HI mouse model, inhalation of high-concentration H_2_ improved the neurological outcome and reduced infarct size and oedema after cerebral ischaemia/reperfusion, even though H_2_ acts independently of antinecroptosis pathways^19^.

H_2_ has been extensively studied under various physiological and pathological conditions. It can be used as an effective antioxidant; owing to its small molecular size, it rapidly diffuses across membranes and can reach and react with cytotoxic free radicals, thus protecting against oxidative damage^15^. Adult rats with cerebral ischaemia showed markedly decreased oxidative stress and suppressed brain injury related to a reduction in microglia when H_2_ was inhaled. Microglia is known for its role in neural inflammation and remodelling^15^.

Although the exact mechanism underlying the interaction of molecular H_2_ with the signalling pathways responsible for neuroprotection is unclear, most previous human and animal studies evaluated its effectiveness by using biomarkers of oxidative stress, inflammation, and apoptosis^16^. However, the effective concentration differed, ranging from 1.3% to 4.0% as ventilatory gas^15,18,20–23^ (Table 1). In our model, ventilation through an endotracheal tube was chosen because most HIE neonates require intensive care and sedation is required during TH. The H_2_ concentration in this study was between 2.1% and 2.7% because 2% H_2_ was the minimal concentration that showed significant neuroprotection in ischaemic rats^15^. The next step should be focused on the optimal concentration and duration of H_2_ therapy and how much of the molecular H_2_ is actually delivered to the organs.

Regarding safety, H_2_ ventilation did not affect the physiological parameters in this study. There were no significant changes in the vitals of piglets in the TH-H_2_ group compared with the TH group. The safety of H_2_ was validated in previous human and animal studies. In patients with post-cardiac arrest syndrome (PCAS), inhalation of 2% H_2_ with oxygen for 18 h did not interfere with PCAS care and no adverse effects due to H_2_ ventilation were observed for 7 days after cardiac arrest compared with conventional PCAS care^17^. Due to its selectivity, H_2_ does not react with other reactive oxygen species with physiological roles^15^. Compared with other neuroprotective agents, H_2_ is widely available and cheaper with fewer adverse effects.

In this study, neurological improvement was also seen in piglets treated with TH alone because we were able to initiate TH as soon as possible within 6 h after the insult (possible latent phase) and maintained it for 24 h. The timing of the TH initiation is particularly important for effective neuroprotection. This is supported by a study showing that early antioxidant treatment using EUK-134 [manganese 3-methoxy N,N′- bis (salicylidene) ethylenediamine chloride] with TH delayed until 4 h after HI had no additive neuroprotection in 4–7-day-old piglets^24^. In actual clinical settings, immediate therapy after rescue is not always feasible. However, the results of our study were intended to be applied to the resuscitation of newborns with asphyxia in institutions where TH is available. Thus, it can fulfil the key requirements of the effective neuroprotection induced by TH. Additionally, TH inhibited apoptotic cell death, not necrosis, after moderate hypoxia-ischaemia in piglets^25^. In our study, the TH-H_2_ group showed fewer TUNEL (+) cells in the cortex in all three examined regions compared with the NT group. Cell death was also significantly reduced in the dorsal cortex compared with the TH group. Such an improvement in TUNEL staining is similar to the results of previous combination therapies such as TH with melatonin and argon gas^26,27^. Thus, a possible improvement in motor function in piglets in the TH-H_2_ group could be due not only to the areas responsible for motor function, but also to the other multiple pathways connecting the various parts of the brain.

In this study, there was no correlation between TUNEL (+) cells in the cortex and the neurological score. We speculate that the possible mechanisms underlying HI followed by reperfusion-induced organ damage are multifactorial and interdependent, involving hypoxia, inflammatory responses, and free radical damage. Thus, we speculate that the neurological improvement in our study is likely due to H_2_ augmentation of the neuroprotection of TH through reduced delayed cell death via a suppression of oxidative stress and inflammation.

One advantage of this study is that we could observe the trajectory of neurological recovery in a large-sized neonatal HIE animal model for 5 days after the insult. For the study of neonatal HIE, piglet models are well established with data on cerebral processes and histological analysis due to similarities in the timing of the brain growth spurt and neuroanatomy around term gestation in human neonates and piglets^28,29^. However, on the other hand, piglets mature rapidly after birth and long-term neurological outcomes are thus not well established in the neonatal period.

The HI insult protocol of our institution is also unique. By using cerebral hemodynamics and EEG as a guide to control the insult, higher survival rates of HI piglets with a considerable amount of brain injury was achieved compared with EEG alone. TRS measures the absolute value of cerebral blood volume in real time and is non-invasive and easy-to-use, which could be useful in bedside examinations^30–33^. By using the above protocol, we were also able to reproduce the symptoms of perinatal asphyxia found in human neonates with comparable physiological and biochemical data among all three groups.

Several limitations of this work are recognised, including technical difficulties for the clinical application of H_2_ ventilation combined with TH in neonates. First, evaluation of the target severity of the HI insult for TH-H_2_ is an important problem. We believe that combined assessment using aEEG and cerebral blood volume can be useful^30^, although several studies in the clinical situation are needed to assess the severity of the HI injury. Hence, further studies are required before the clinical application of H_2_ to (1) determine the suitable parameters defining the target group for TH-H_2_ in the immediate transition after birth, (2) evaluate the most appropriate concentrations of H_2_ or whether an increased duration of ventilation combined with TH would provide a greater degree of protection, and (3) develop a device for continuous delivery of H_2_ gas that can also be connected to neonatal ventilators.

Due to the complexity of the neuroprotective pathways involved, a great deal of time and effort is required to better understand H_2_ medicine in neonatal HIE.

To conclude, H_2_ ventilation combined with TH improves the short-term neurological function of HI neonatal piglets by boosting the neuroprotection afforded by TH, presumably through a reduction in delayed cell death via suppression of oxidative stress and inflammation.

## Materials and Methods

### Ethical approval and animal preparation

The study protocol was approved by the Animal Care and Use Committee for Kagawa University (15070-1) and in accordance with Animal Research: Reporting *In Vivo* Experiments guidelines. Twenty-four newborn piglets within 24 h after birth (17 males, 7 females; body weight ranging from 1530 to 2150 g) were anaesthetised and surgically prepared.

Before the experimental procedures, the piglets were placed under a radiant warmer and their activities and alertness briefly observed. Anaesthesia was induced with 1–2% isoflurane (Forane® inhalant liquid; Abbott Co., Tokyo, Japan) in air using a facemask. Each piglet was then intubated and mechanically ventilated with an infant ventilator. The umbilical vein and artery were cannulated with a 3- or 4-Fr neonatal umbilical catheter (Atom Indwelling Feeding Tube for Infants; Atom Medical Co., Tokyo, Japan); the umbilical vein catheter was at a site 5 cm in depth from the incision for blood pressure monitoring, and the umbilical artery catheter was at a site 15 cm in depth from the incision for blood sampling. After cannulation, the piglets were anaesthetised with fentanyl citrate at an initial dose of 10 µg/kg followed by continuous infusion at 5 µg/kg/h and were paralysed with pancuronium bromide at an initial dose of 100 µg/kg followed by continuous infusion at 100 µg/kg/h. Maintenance solution (electrolytes plus 2.7% glucose [KN3B]; Otsuka Pharmaceutical Co., Tokyo, Japan) was infused continuously at a rate of 4 mL/kg/h via the umbilical vein (glucose was infused at a rate of 2 mg/kg/min). Arterial blood samples were taken at critical points and when clinically indicated throughout the experiment. Each piglet was then placed in a copper mesh-shielded cage under a radiant warmer to maintain a rectal temperature of 38.0 ± 0.5 °C. Inspired gas was prepared by mixing O_2_ and N_2_ gases to obtain the oxygen concentrations required for the experiment. Ventilation was adjusted to maintain PaO_2_ and PaCO_2_ within their normal ranges. Arterial blood pressures were measured and recorded via the umbilical arterial catheter.

### Time-resolved near-infrared spectroscopy and analysis

A portable three-wavelength TRS system (TRS-10; Hamamatsu Photonics K.K., Hamamatsu, Japan) was applied using probes attached to the head of each piglet. The light emitter and detector optodes were positioned on the parietal region of each piglet with a 30-mm interoptode distance. In the TRS system, a time-correlated single-photon-counting technique is used for detection. The concentrations of oxyhaemoglobin (oxyHb) and deoxyhaemoglobin (deoxyHb) were calculated from the absorption coefficients of oxyHb and deoxyHb, with the assumption that background absorption was due only to 85% (by volume) water. The total cerebral Hb concentration (totalHb), ScO_2_, and cerebral blood volume were calculated as described previously^34,35^.

### Amplitude-integrated electroencephalography

Neural activity was measured by aEEG (Nicolet One; Cardinal Health, Inc., Dublin, OH). All electrical devices and the copper mesh shield were grounded. The signal was displayed on a semi-logarithmic scale at a low speed (6 cm/h). Measurements were conducted every second. Gold-plated electrode needles were placed at the P3 and P4 positions, which corresponded to the left and right parietal regions of the head. The maximum amplitude <5 µV was defined as LAEEG.

### Hypoxic-ischaemic insult protocol

**Figure 6:**
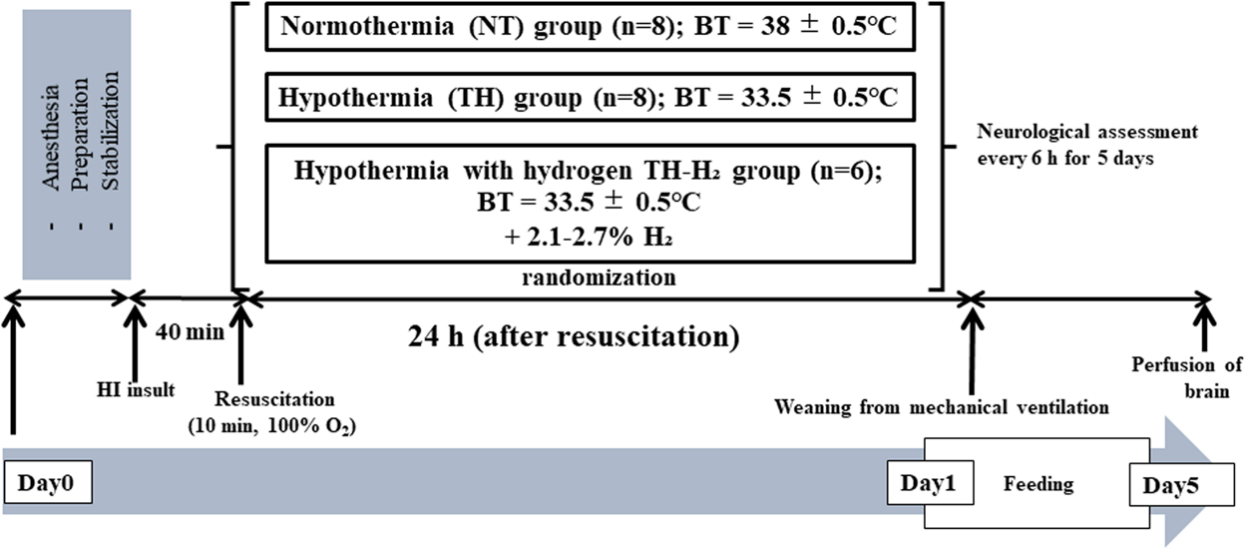
Experimental timeline. After surgical preparation and stabilization, a hypoxic-ischaemic insult was induced, followed by resuscitation. Piglets were then randomised into normothermia (NT), hypothermia (TH), and TH with hydrogen ventilation (TH-H_2_) groups. From 24 h after the insult, piglets were weaned from mechanical ventilation, feeding was initiated, and neurological assessments were performed. At day 5 post-insult, brains were harvested for histopathological analysis.

Because the details were reported in our previous studies^30,31^, only an outline of the HI insult protocol is provided (Fig. 6). Hypoxia was induced by reducing the inspired oxygen concentration of the ventilator to 4% after at least 120 min of stabilization from the initial anaesthetic induction. To obtain an LAEEG pattern (<5 µV), the inspired oxygen concentration was reduced further if required, adjusting it so as to not cause cardiopulmonary arrest. From the beginning of LAEEG, the insult was continued for 30 min. FiO_2_ was decreased (1% decrements) or increased (1% increments) during the insult to maintain the LAEEG, HR (>130 beats/min), and MABP (>70% of baseline). LAEEG was maintained for 20 min. For the final 10 min of the 30-min insult, if the MABP exceeded 70% of the baseline, hypotension was induced by decreasing the FiO_2_. Resuscitation was performed when the cerebral blood volume value dropped below 30% and/or the MABP declined below 70% of baseline. Hypoxia was terminated by resuscitation with 100% oxygen. NaHCO_3_ was used to correct a base deficit (base excess below −5.0 mEq/L) to maintain a pH of 7.3–7.5. After 10 min of 100% FiO_2_, the ventilator rate and FiO_2_ were gradually reduced to maintain an SpO_2_ of 95–98%.

### Post-insult treatment

After the HI insult, 24 piglets were randomised into three groups: HI insult with normothermia (NT, n = 9), HI insult with TH (TH, 33.5 ± 0.5 °C, n = 8) and insult with TH with H_2_ ventilation (TH-H_2_, 2.1–2.7% H_2_, n = 7). Whole-body hypothermia was achieved using a cooling blanket (Medicool; MAC8 Inc., Tokyo, Japan) after resuscitation. The piglets were cooled to 33.5 ± 0.5 °C for 24 h and then rewarmed at 1 °C/h using a blanket. The rectal temperature was used as the measure of body temperature. The temperature of the incubator was maintained at 28–32 °C. Once the piglets were weaned off the anaesthesia and ventilator and extubated, they were allowed to recover and were maintained for 5 days in the incubator. Piglets were fed 50–100 mL artificial animal milk via a nasogastric tube every 6 h. The presence of seizures was recognised clinically as rhythmic pathologic movements (cycling) and tonic postures sustained between cycling episodes. If seizures occurred, the piglet was treated with phenobarbital (20 mg/kg) via intramuscular injection. If seizures persisted, the piglet was treated with two successive anticonvulsant doses. If seizures persisted after two successive anticonvulsant doses, the piglet was euthanised.

**Figure 7:**
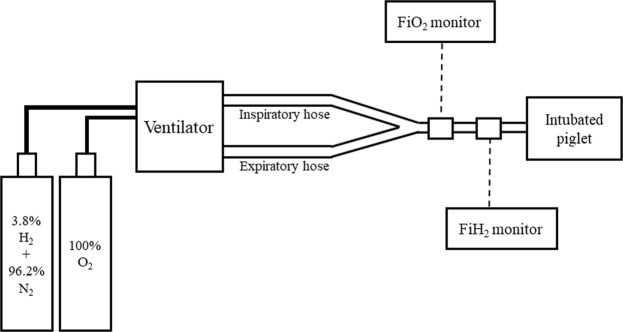
Hydrogen gas therapy. Two gas cylinders—one containing a gas mixture of 3.8% H_2_ and 96.2% N_2_, the other containing 100% O_2_—were connected to the ventilator. The concentration of H_2_ gas was measured by a portable gas monitor (FiGX-8000, RIKEN KEIKI Co., Ltd., Tokyo, Japan). The H_2_ concentration was maintained between 2.1% and 2.7% according to the O_2_ requirements of each piglet (FiO_2_ 0.21–0.4). After hydrogen therapy, the cylinder was replaced with an air compressor again.

For H_2_ inhalation, two types of cylinders were used: one contained a gas mixture containing 3.8% H_2_ and 96.2% N_2_; the other contained 100% O_2_, as shown in Fig. 7. The H_2_ concentration depended on the oxygen requirement of each piglet. Therefore, the H_2_ concentration was usually between 2.1 and 2.7 (FiO_2_ range, 0.21–0.4) during the therapy. H_2_ gas was delivered through the ventilator for 24 h. The concentration of H_2_ gas was measured by a portable gas monitor (GX-8000, RIKEN KEIKI Co., Ltd., Japan). After 24 h of treatment, the hydrogen-nitrogen gas mixture was replaced with an air compressor again (Fig. 7).

For piglets given TH, their temperature was automatically controlled to maintain the target temperature (rectal temperature, 33–34 °C) during TH and rewarmed at the rate of 1 °C/h by a cooling blanket. Anaesthesia was stopped at the beginning of the rewarming period. For NT piglets, the rectal temperature was monitored continuously to maintain a normal range (38–39 °C) under the radiant warmer under anaesthesia-ventilation during 24 h after the insult. The anaesthesia was then stopped, followed by extubation.

### Neurological assessment

Soon after the piglets were nursed in the incubator, neurological function was observed by examiners who were blinded to the protocols. Neurological examination was carried out every 6 h for 5 days from day 1 to day 5 post-insult. The neurological scoring comprised nine neurologic items: a, respiration; b, consciousness; c, orientation; d, ability to walk; e, ability to control the forelimbs; f, ability to control the hind limbs; g, maintenance of tone; and h, pathological movements (scored as: 2, normal; 1, moderately abnormal; or 0, definitely pathologic). The minimum score is 0 and the maximum is 18, indicating a normal healthy piglet^36^.

### Histological assessment

On day 5 after the insult, the brain of each animal was perfused with 0.9% saline and 4% phosphate-buffered paraformaldehyde. Histological evaluations of brain tissue were performed, and irregularities were graded according to a histopathology grading scale for a piglet model of posthypoxic encephalopathy, which has also been validated^36,37^. Coronal blocks of the gray matter, white matter, hippocampus, and cerebellum were embedded in paraffin and cut with a microtome at 4 μm. At regular intervals, three sections of each sample were examined. For H&E staining, the extent of damage in each of the four regions was graded in 0.5-unit intervals on a 9-step scale that ranged from 0.0–4.0. Grade 0 indicated no damage; grade 1 indicated ≤10% of the area affected with morphological changes that included individual necrotic neurons and small patchy, complete or incomplete infarcts; grade 2 indicated 20–30% of the area affected with partly confluent incomplete or complete infarcts; grade 3 indicated 40–60% of the area affected with large confluent and complete infarcts; and grade 4 indicated >75% of the area affected with neuronal necrosis in the hippocampus and the total disintegration of the cortex^36^.

**Figure 8:**
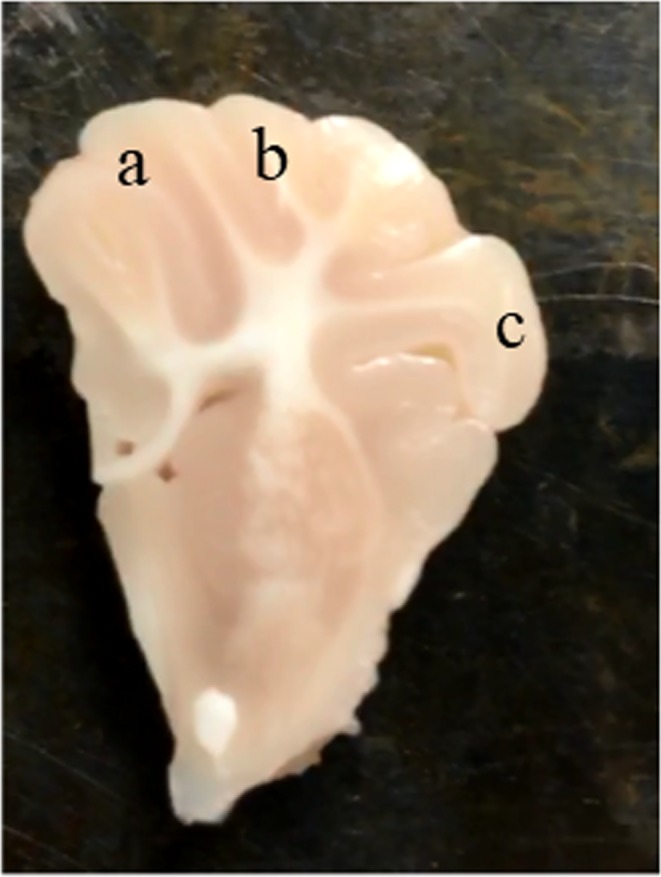
Representative piglet brain photograph indicating brain regions assessed for TUNEL-positive cells in the cerebral cortex. Region a indicates the dorsal cortex, b indicates the sensorimotor cortex, and c indicates the mid-temporal cortex.

TUNEL assays were performed with an ApopTag Plus Peroxidase *In Situ* Apoptosis Detection Kit (ApopTag®, EMD Millipore Corp., Burlington, MA) as instructed by the manufacturer’s protocol. TUNEL (+) cells were counted in three areas of the cortical gray matter—dorsal cortex (DCx), sensorimotor cortex (SMCx), and mid-temporal cortex (MTCx)—under high magnification (Figs 3 and 8). Areas were determined according to the previous reports^26,27^.

### Data analysis

GraphPad Prism 7.02 (GraphPad Software, La Jolla, CA) was used for all statistical analyses. Piglets that died were excluded from the statistical analysis. The final total sample size was 22 (NT, 8; TH, 8; and TH-H_2_, 6). Values are expressed as the mean ± SD for physiological and blood gas data, duration of LAEEG, and time until the target temperature in the TH and TH-H_2_ groups. For the neurological score, histological score and TUNEL (+) cell counting, the median with interquartile range was used. Physiological data, blood gas data, total duration of LAEEG were compared among the three groups at each time point (baseline and 0, 1, 6, 12, and 24 h after the insult). For the comparison of each time point with the baseline value, Dunnett’s multiple comparisons test was used. The percentage of piglets that could walk on day 3 was compared using the chi-square test. For the neurological score, histological score, and TUNEL cell counting, one-way analysis of variance followed by Tukey’s multiple comparison test was used. A p value of <0.05 was considered significant.

## Supplementary information

Normal and abnormal patterns of walking
